# Supplementary material for: Iterative improvement in the automatic modular design of robot swarms
Source: PeerJ Comput Sci. 2020 Dec 7;6:e322. doi: 10.7717/peerj-cs.322 (PMC7924708; doi:10.7717/peerj-cs.322)
Supplement: Supplemental Information 3 [file peerj-cs-06-322-s003.zip › argos3/doc/api/standalone/a00631_source.html]

ARGoS: plugins/simulator/visualizations/qt-opengl/models/qtopengl\_cylinder.cpp Source File


- Main Page
- Related Pages
- Namespaces
- Classes
- Files

- File List
- File Members

# plugins/simulator/visualizations/qt-opengl/models/qtopengl\_cylinder.cpp

Go to the documentation of this file.

```
00001 
00007 #include "qtopengl_cylinder.h"
00008 #include <argos3/core/utility/math/vector2.h>
00009 #include <argos3/plugins/simulator/entities/led_equipped_entity.h>
00010 #include <argos3/plugins/simulator/entities/cylinder_entity.h>
00011 #include <argos3/plugins/simulator/visualizations/qt-opengl/qtopengl_widget.h>
00012 
00013 namespace argos {
00014 
00015    /****************************************/
00016    /****************************************/
00017 
00018    static const Real LED_RADIUS = 0.01f;
00019    const GLfloat MOVABLE_COLOR[]    = { 0.0f, 1.0f, 0.0f, 1.0f };
00020    const GLfloat NONMOVABLE_COLOR[] = { 0.7f, 0.7f, 0.7f, 1.0f };
00021    const GLfloat SPECULAR[]         = { 0.0f, 0.0f, 0.0f, 1.0f };
00022    const GLfloat SHININESS[]        = { 0.0f                   };
00023    const GLfloat EMISSION[]         = { 0.0f, 0.0f, 0.0f, 1.0f };
00024 
00025    /****************************************/
00026    /****************************************/
00027 
00028    CQTOpenGLCylinder::CQTOpenGLCylinder() :
00029       m_unVertices(20) {
00030 
00031       /* Reserve the needed display lists */
00032       m_unBaseList = glGenLists(1);
00033       m_unBodyList = m_unBaseList;
00034       m_unLEDList = m_unBaseList + 1;
00035 
00036       /* Make body list */
00037       glNewList(m_unBodyList, GL_COMPILE);
00038       MakeBody();
00039       glEndList();
00040 
00041       /* Make LED list */
00042       glNewList(m_unLEDList, GL_COMPILE);
00043       MakeLED();
00044       glEndList();
00045    }
00046 
00047    /****************************************/
00048    /****************************************/
00049 
00050    CQTOpenGLCylinder::~CQTOpenGLCylinder() {
00051       glDeleteLists(m_unBaseList, 2);
00052    }
00053 
00054    /****************************************/
00055    /****************************************/
00056 
00057    void CQTOpenGLCylinder::DrawLEDs(CCylinderEntity& c_entity) {
00058       /* Draw the LEDs */
00059       GLfloat pfColor[]           = {   0.0f, 0.0f, 0.0f, 1.0f };
00060       const GLfloat pfSpecular[]  = {   0.0f, 0.0f, 0.0f, 1.0f };
00061       const GLfloat pfShininess[] = { 100.0f                   };
00062       const GLfloat pfEmission[]  = {   0.0f, 0.0f, 0.0f, 1.0f };
00063       glMaterialfv(GL_FRONT_AND_BACK, GL_SPECULAR, pfSpecular);
00064       glMaterialfv(GL_FRONT_AND_BACK, GL_SHININESS, pfShininess);
00065       glMaterialfv(GL_FRONT_AND_BACK, GL_EMISSION, pfEmission);
00066       CLEDEquippedEntity& cLEDEquippedEntity = c_entity.GetLEDEquippedEntity();
00067       for(UInt32 i = 0; i < cLEDEquippedEntity.GetLEDs().size(); ++i) {
00068          glPushMatrix();
00069          /* Set the material */
00070          const CColor& cColor = cLEDEquippedEntity.GetLED(i).GetColor();
00071          pfColor[0] = cColor.GetRed()   / 255.0f;
00072          pfColor[1] = cColor.GetGreen() / 255.0f;
00073          pfColor[2] = cColor.GetBlue()  / 255.0f;
00074          glMaterialfv(GL_FRONT_AND_BACK, GL_AMBIENT_AND_DIFFUSE, pfColor);
00075          /* Perform rototranslation */
00076          const CVector3& cPosition = cLEDEquippedEntity.GetLEDOffset(i);
00077          glTranslatef(cPosition.GetX(), cPosition.GetY(), cPosition.GetZ());
00078          /* Draw the LED */
00079          glCallList(m_unLEDList);
00080          glPopMatrix();
00081       }
00082    }
00083 
00084    /****************************************/
00085    /****************************************/
00086 
00087    void CQTOpenGLCylinder::Draw(CCylinderEntity& c_entity) {
00088       /* Draw the body */
00089       if(c_entity.GetEmbodiedEntity().IsMovable()) {
00090          glMaterialfv(GL_FRONT_AND_BACK, GL_AMBIENT_AND_DIFFUSE, MOVABLE_COLOR);
00091       }
00092       else {
00093          glMaterialfv(GL_FRONT_AND_BACK, GL_AMBIENT_AND_DIFFUSE, NONMOVABLE_COLOR);
00094       }
00095       glPushMatrix();
00096       glScalef(c_entity.GetRadius(), c_entity.GetRadius(), c_entity.GetHeight());
00097       glCallList(m_unBodyList);
00098       glPopMatrix();
00099    }
00100 
00101    /****************************************/
00102    /****************************************/
00103 
00104    void CQTOpenGLCylinder::MakeBody() {
00105       /* Since this shape can be stretched,
00106          make sure the normal vectors are unit-long */
00107       glEnable(GL_NORMALIZE);
00108 
00109       /* Set the material */
00110       glMaterialfv(GL_FRONT_AND_BACK, GL_SPECULAR, SPECULAR);
00111       glMaterialfv(GL_FRONT_AND_BACK, GL_SHININESS, SHININESS);
00112       glMaterialfv(GL_FRONT_AND_BACK, GL_EMISSION, EMISSION);
00113 
00114       /* Let's start the actual shape */
00115       /* Side surface */
00116       CVector2 cVertex(1.0f, 0.0f);
00117       CRadians cAngle(CRadians::TWO_PI / m_unVertices);
00118       glBegin(GL_QUAD_STRIP);
00119       for(GLuint i = 0; i <= m_unVertices; i++) {
00120          glNormal3f(cVertex.GetX(), cVertex.GetY(), 0.0f);
00121          glVertex3f(cVertex.GetX(), cVertex.GetY(), 1.0f);
00122          glVertex3f(cVertex.GetX(), cVertex.GetY(), 0.0f);
00123          cVertex.Rotate(cAngle);
00124       }
00125       glEnd();
00126       /* Top disk */
00127       cVertex.Set(1.0f, 0.0f);
00128       glBegin(GL_POLYGON);
00129       glNormal3f(0.0f, 0.0f, 1.0f);
00130       for(GLuint i = 0; i <= m_unVertices; i++) {
00131          glVertex3f(cVertex.GetX(), cVertex.GetY(), 1.0f);
00132          cVertex.Rotate(cAngle);
00133       }
00134       glEnd();
00135       /* Bottom disk */
00136       cVertex.Set(1.0f, 0.0f);
00137       cAngle = -cAngle;
00138       glBegin(GL_POLYGON);
00139       glNormal3f(0.0f, 0.0f, -1.0f);
00140       for(GLuint i = 0; i <= m_unVertices; i++) {
00141          glVertex3f(cVertex.GetX(), cVertex.GetY(), 0.0f);
00142          cVertex.Rotate(cAngle);
00143       }
00144       glEnd();
00145       /* The shape definition is finished */
00146 
00147       /* We don't need it anymore */
00148       glDisable(GL_NORMALIZE);
00149 
00150    }
00151 
00152    /****************************************/
00153    /****************************************/
00154 
00155    void CQTOpenGLCylinder::MakeLED() {
00156       CVector3 cNormal, cPoint;
00157       CRadians cSlice(CRadians::TWO_PI / m_unVertices);
00158 
00159       glBegin(GL_TRIANGLE_STRIP);
00160       for(CRadians cInclination; cInclination <= CRadians::PI; cInclination += cSlice) {
00161          for(CRadians cAzimuth; cAzimuth <= CRadians::TWO_PI; cAzimuth += cSlice) {
00162 
00163             cNormal.FromSphericalCoords(1.0f, cInclination, cAzimuth);
00164             cPoint = LED_RADIUS * cNormal;
00165             glNormal3f(cNormal.GetX(), cNormal.GetY(), cNormal.GetZ());
00166             glVertex3f(cPoint.GetX(), cPoint.GetY(), cPoint.GetZ());
00167 
00168             cNormal.FromSphericalCoords(1.0f, cInclination + cSlice, cAzimuth);
00169             cPoint = LED_RADIUS * cNormal;
00170             glNormal3f(cNormal.GetX(), cNormal.GetY(), cNormal.GetZ());
00171             glVertex3f(cPoint.GetX(), cPoint.GetY(), cPoint.GetZ());
00172 
00173             cNormal.FromSphericalCoords(1.0f, cInclination, cAzimuth + cSlice);
00174             cPoint = LED_RADIUS * cNormal;
00175             glNormal3f(cNormal.GetX(), cNormal.GetY(), cNormal.GetZ());
00176             glVertex3f(cPoint.GetX(), cPoint.GetY(), cPoint.GetZ());
00177 
00178             cNormal.FromSphericalCoords(1.0f, cInclination + cSlice, cAzimuth + cSlice);
00179             cPoint = LED_RADIUS * cNormal;
00180             glNormal3f(cNormal.GetX(), cNormal.GetY(), cNormal.GetZ());
00181             glVertex3f(cPoint.GetX(), cPoint.GetY(), cPoint.GetZ());
00182 
00183          }
00184       }
00185       glEnd();
00186    }
00187 
00188    /****************************************/
00189    /****************************************/
00190 
00191    class CQTOpenGLOperationDrawCylinderNormal : public CQTOpenGLOperationDrawNormal {
00192    public:
00193       void ApplyTo(CQTOpenGLWidget& c_visualization,
00194                    CCylinderEntity& c_entity) {
00195          static CQTOpenGLCylinder m_cModel;
00196          c_visualization.DrawEntity(c_entity.GetEmbodiedEntity());
00197          m_cModel.Draw(c_entity);
00198          m_cModel.DrawLEDs(c_entity);
00199       }
00200    };
00201 
00202    class CQTOpenGLOperationDrawCylinderSelected : public CQTOpenGLOperationDrawSelected {
00203    public:
00204       void ApplyTo(CQTOpenGLWidget& c_visualization,
00205                    CCylinderEntity& c_entity) {
00206          c_visualization.DrawBoundingBox(c_entity.GetEmbodiedEntity());
00207       }
00208    };
00209 
00210    REGISTER_QTOPENGL_ENTITY_OPERATION(CQTOpenGLOperationDrawNormal, CQTOpenGLOperationDrawCylinderNormal, CCylinderEntity);
00211 
00212    REGISTER_QTOPENGL_ENTITY_OPERATION(CQTOpenGLOperationDrawSelected, CQTOpenGLOperationDrawCylinderSelected, CCylinderEntity);
00213 
00214    /****************************************/
00215    /****************************************/
00216 
00217 }
```

---

Generated on 10 Jul 2018 for ARGoS by 
 1.6.1
